# Periodontal Biological Events Associated with Orthodontic Tooth Movement: The Biomechanics of the Cytoskeleton and the Extracellular Matrix

**DOI:** 10.1155/2015/894123

**Published:** 2015-08-13

**Authors:** L. Feller, R. A. G. Khammissa, I. Schechter, A. Moodley, G. Thomadakis, J. Lemmer

**Affiliations:** ^1^Department of Periodontology and Oral Medicine, Sefako Makgatho Health Sciences University, Pretoria 0204, South Africa; ^2^Schulich Faculty of Chemistry, Technion-Israel Institute of Technology, 32000 Haifa, Israel; ^3^Private Practice, 15 School Road, Morningside, Johannesburg 2057, South Africa

## Abstract

The mechanical stimuli generated by orthodontic forces cause deformation of extracellular matrices and cells, vascular changes, inflammation, and the release of active biological agents generating a complex multifactorial sequence of biological events culminating in bone remodelling enabling orthodontic tooth movement. Orthodontic forces on the teeth generate stresses in periodontal tissues according to a number of variables including the type (continuous, interrupted, or intermittent), magnitude, direction, and frequency of the applied load. Whether the strain is compressive or tensile determines whether bone deposition or bone resorption will occur. The mechanically induced strains mediate structural changes in extracellular matrices and in cells, consequently affecting cellular gene expression and function. In the extracellular matrix, mechanosensing molecules integrated into the structure of various proteins can be activated upon load-induced protein unfolding. These specialized molecules have the capacity to sense and then to convert microenvironmental biomechanical stimuli into intracellular biochemical signals that interact to generate a coordinated tissue response. It is also possible that the applied force may directly cause nuclear deformation with configurational changes in chromatin, thus influencing gene expression. In this review article we summarize the current general concepts of mechanotransduction influencing the remodelling of periodontal tissues thus enabling tooth movement in response to applied orthodontic loads.

## 1. Introduction

Orthodontic forces applied to teeth generate complex mechanical loading patterns comprising compressive, tensile, and shear strains which in turn elicit diverse and complex biological responses in the periodontal tissues immediately surrounding the loaded teeth [1]. These mechanically induced tissue strains are determined by a number of variables including the type of force (i.e., continuous, interrupted, and intermediate), the magnitude, direction, location, and frequency of the load applied [1, 2]; the shapes, number, lengths, locations, and angulations of the mechanically loaded teeth [3]; and the material properties of the local periodontal ligament and alveolar bone [3, 4].

In the context of orthodontic tooth movement, for purposes of convenience, one can identify two distinct zones in the periodontal ligament and associated alveolar bone: a zone where the periodontal ligament and adjacent alveolar bone are compressed and a zone where the tissues are under tension [1, 2, 5]. Although it is an oversimplification, the response to applied orthodontic forces is bone formation on the tension side and bone resorption on the compression side [1, 2, 5]. The net outcome of this tissue remodelling is tooth movement [2, 6].

In general, compressive and tensile stresses induce both directly and indirectly the release of specific active signaling molecules/biological agents by a variety of local cells including fibroblasts, macrophages, cementoblasts, osteoblasts, osteoclasts, and cells of the local vasculature [1, 7]. These specific agents are differentially expressed at various sites around the mechanically loaded teeth and mediate the dynamic tissue responses involved in tooth movement.

However, despite this differential expression of tissue remodelling agents, it appears that, as in other complex systems, the level of expression of a specific agent and its interaction with other biologically active agents in the microenvironment, rather than merely the type of the agent, are important in determining the biological outcomes of bone resorption and bone formation [1]. A complex system of diverse interacting agents will have many interactions, the behaviour of each agent influenced by the other agents. The aggregate of the integrated activity of the several interacting biological agents is not one of simple cause and effect behavior and therefore cannot be derived from summation of the activity of the individual agents [8–10].

The biologically active agents released into the local microenvironment by the cells mentioned above in response to load-induced strains comprise cytokines, neurotransmitters, matrix metalloproteinases, growth factors, and bone proteins including tumour necrosis factor-*α* (TNF-*α*), receptor activator of nuclear factor-*κ*B ligand (RANKL), osteoprotegerin (OPG), interleukin-10 (IL-10), IL-6, IL-1*β*, endothelins, prostaglandins, osteocalcin, vascular endothelial growth factor (VEGF), macrophage-colony stimulating factor (M-CSF), osteopontin, and collagen 1 [1, 5–7, 11, 12]. Some of these active agents are proinflammatory mediators generating a transitional, aseptic inflammatory response triggering the process of tissue remodelling. An inflammatory element is thus essential for orthodontic tooth movement [1, 11, 13, 14].

## 2. Stages of Orthodontic Tooth Movement

In general it appears that greater orthodontic forces generating higher magnitudes of stresses promote significant tissue necrosis and hyalinization [12, 15], and tooth movement can occur only at the expense of undermining bone resorption and a hypoxically degenerated periodontal ligament [11, 16]. On the other hand, lower orthodontic forces generating lower magnitudes of stress are associated with little or no tissue necrosis and hyalinization. Under these circumstances, direct frontal resorption of the alveolar bone and remodelling of the vascular and fibrous elements of the periodontal ligament will allow continuous steady tooth movement with no significant lag phase [12, 15]. However, in practice, some degree of undermining resorption is unavoidable.

This said, three stages of orthodontic tooth movement have been described. Stage one (initial stage) lasts about 24 to 48 hours and is characterized by tooth displacement in the periodontal ligament space within its bone socket. Stage two (lag stage) lasts 20–30 days and is characterized by the formation of necrosis and hyalinization in response to compression of the vasculature and subsequent hypoxia in the periodontal ligament and adjacent alveolar bone [5, 11, 16–19]. The hyalinized tissue is not only found in the compression zone ahead of the moving tooth but also lingually and buccally caused by local morphological variations of tooth and alveolar bone [16]. The hyalinized tissue is removed by macrophages and multinucleated giant cells and the necrotic bone by undermining resorption [16, 19, 20]. In this lag stage there is little or no tooth movement [5, 17].

In stage 3, the postlag stage, there is tooth movement mediated by bone remodelling through the agency of osteoclasts and osteoblasts on a background of neoangiogenesis. The neoangiogenesis is predominantly mediated by hypoxia-inducible factor-1 (HIF-1) and VEGF, but also by other biological agents including fibroblast growth factor (FGF), TNF*α*, and transforming growth factor (TGF)-*β* [5, 19, 20].

There is substantial variation in the rate of tooth movement in different but comparable subjects in response to apparently identical orthodontic forces. This strongly suggests that polymorphism of genes encoding cytokines and other subject-specific factors influence the biological responses to similar orthodontic forces [15].

## 3. Stress, Strain, and the Cytoskeleton

In continuum mechanics, deformation is defined as transformation of a material body (e.g., the position of all body particles) from a reference configuration to its current configuration [21]. Strain is a measure of deformation representing the displacement of particles in the body relative to their reference locations. Deformations which revert to the reference configuration after the stresses have been removed are termed elastic deformations while plastic, viscous, and desmoplastic deformations are irreversible and occur when the stresses applied exceed the elastic limit of the body material [22]. In this regard, viscoelastic material is one that displays both viscous and elastic properties when it undergoes deformation. On the other hand stiffness can be defined as the resistance to deformation exhibited by an elastic body material in response to the applied force [22, 23], while the reverse of the stiffness is termed compliance or flexibility [24].

The cytoskeleton is a dynamic structure comprising three main polymeric elements, microtubules, actin filaments, and intermediate filaments, organized in a network that provides resistance to stress-induced deformation. The architecture of the structure determines the mechanical behaviour of the cytoskeleton and the physical properties of the cell. The cytoskeleton generates, transmits, and responds to both internal and external mechanical stresses, and through the cytoskeletal network mechanical stimuli mediate cell shape, motility, differentiation, survival, and ultimately cell behaviour. In response to applied forces, the cytoskeletal network stiffens, thus resisting additional deformation, and reorganizes in order to maintain the cellular integrity [25].

The intermediate filaments are the least stiff of the cytoskeletal components and therefore withstand tensile and sheer stresses more efficiently than do microtubules and actin filaments [26]. In response to mechanical stresses, assembly and integration of additional intermediate filaments take place, which then interconnect with each other and with actin filaments and microtubules, contributing to the structural integrity of the cell [25].

On the other hand, the microtubules are by far the stiffest cytoskeletal component [25] and therefore cannot withstand large tensile or shear stresses without exhibiting deformation, but they can resist large compressive stresses and thus contribute to the stability of the cytoskeleton and of the cell shape [26].

Although actin filaments are less stiff than microtubules, a high concentration of molecular crosslinks increases their stiffness, and networks of highly branched actin filaments can generate the forces associated with changes in cell shape, and with cell motility [25]. They withstand more efficiently tensile or shear stresses than do microtubules [26]. Cross-bridging interaction between cytoskeletal actin and myosin filaments generate contractile forces which are transmitted to the extracellular matrix as traction forces, causing strains in the extracellular matrix, which consequently may become stiffer [27].

Cells adhere to, and pull on their extracellular matrix in a way that is related to the degree of stiffness of the matrix [27]. Stiff matrices induce strains at the cellular focal adhesions with consequent strong cell adhesion to the extracellular matrix. Conversely, flexible matrices do not have the capacity to generate the necessary required stress at focal adhesion sites for the development of mature fully functional focal adhesions [22]. In this regard, it has been shown that undifferentiated mesenchymal progenitor cells will differentiate into distinct lineages according to the stiffness characteristic of their microenvironmental matrices [22], and these variations in stiffness of the microenvironmental extracellular matrices also influence cell shape [22].

Extracellular mechanical stresses can activate cell membrane integrins and focal adhesion molecules triggering the assembly of contractile actin-filamentary bundles, termed “stress fibers” [25]. These stress fibers form intracellularly at the cell adhesion-extracellular contact sites in response to elevated mechanical stresses, generating contractile forces [26].

The contractile activity of the actin-myosin cytoskeleton is usually inwardly directed towards the center of the cell. This activity may be induced either by extracellular matrix stresses or stiffness, or by intracellular forces. These contractile forces can activate intracellular signaling pathways that regulate gene expression, cell morphology, and ultimately cell function [22].

In the context of orthodontic tooth movement, orthodontic load-induced compressive, tensile or shear stresses cause dynamic alterations within the extracellular matrix and within the cytoskeleton of cells, in the local microenvironment. The stress-induced strain mediates the proliferation, differentiation, motility, and morphology of osteoblasts, osteoclasts, and other cells in the periodontal ligament and in the alveolar bone, as well as the production of cytokines and growth factors. All these together determine the balance between bone resorption and formation, thus ultimately influencing local tissue remodelling that allows tooth movement in response to applied orthodontic loads.

## 4. Extracellular Matrix (ECM) in relation to Orthodontic Tooth Movement

The extracellular matrix is a complex structure of interacting molecules forming a physical microenvironment of a three-dimensional mesh-like fibrous scaffold, and its biochemical makeup, architecture, and mechanical properties are all essential elements contributing to the determination of physiological responses and function and of survival of cells. Cell anchorage and the vector of cell migration are determined by the fibrous element of the extracellular matrix [11, 28–31].

**Figure 1:**
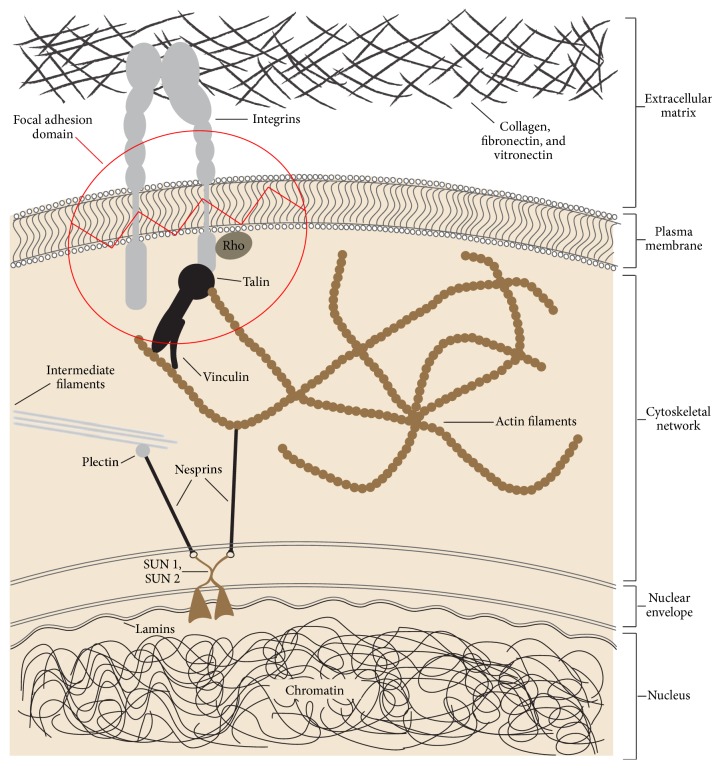
Focal adhesions are protein complexes which connect extracellular matrix proteins such as collagen, fibronectin, and vitronectin to the intracellular actin cytoskeleton by way of integrins and talin. The extracellular matrix derived strain causes configurational changes in the focal adhesion protein talin, exposing vinculin-binding molecular sites. This results in recruitment of vinculin leading to reinforcement of the focal adhesions and establishing a physical link between extracellular matrix and the nuclear envelope through the agency of the integrin-talin/vinculin-actin filament molecular chain. This chain of linked molecules ultimately transmits signals from the mechanically stressed extracellular matrix to the nucleus via the nuclear envelope lamins, nesprins, SUN1, and SUN2. Rho GTPase regulates cytoskeletal organization and in response to extracellular mechanical stimuli, Rho activation promotes myosin contractility and induces the reinforcement of focal adhesions [22, 23, 32].

Cells adhere to the extracellular matrix by focal adhesion domains at the cell plasma membrane. These domains are localised multifunctional dynamic protein complexes comprising transmembrane integrins and cytoplasmic proteins including focal adhesion kinase (FAK), Src, paxillin, tensin, and filamin, linking the cytoskeleton to extracellular matrix proteins including fibronectin, vitronectin, and collagen (Figure 1). These focal adhesion domains mediate reciprocal interactions between the extracellular matrix and the cell and influence the assembly and remodelling of the extracellular matrix on one hand and cell adhesion, migration, proliferation, differentiation, function, and apoptosis on the other hand (Figure 1) [4, 22, 23, 32].

Biochemically stressed extracellular matrix promotes integrin clustering and activation and assembly of focal adhesion proteins and induces Rho pathway-mediated cell contractility. The small G-protein Rho, a member of the Rho family of small guanosine triphosphatases (GTPase), is an important regulator of cytoskeletal tension (Figure 1). Through Rho-associated kinase (ROCK), activated Rho enhances polymerization of myosin light-chains and subsequently cross-bridging interactions between cytoskeletal actin and myosin filaments, mediating the development of stress fibers and increasing cytoskeletal tension and contractility. This in turn upregulates intracellular signalling pathways associated with cell cycle progression and thus promotes cell proliferation. Through the Rho-ROCK pathway, stiff extracellular matrices or external loads further promote formation of stress fibers, integrin clustering, and focal adhesion reinforcement, with a positive impact on cell-matrix adhesion [22, 27, 32–34].

**Figure 2:**
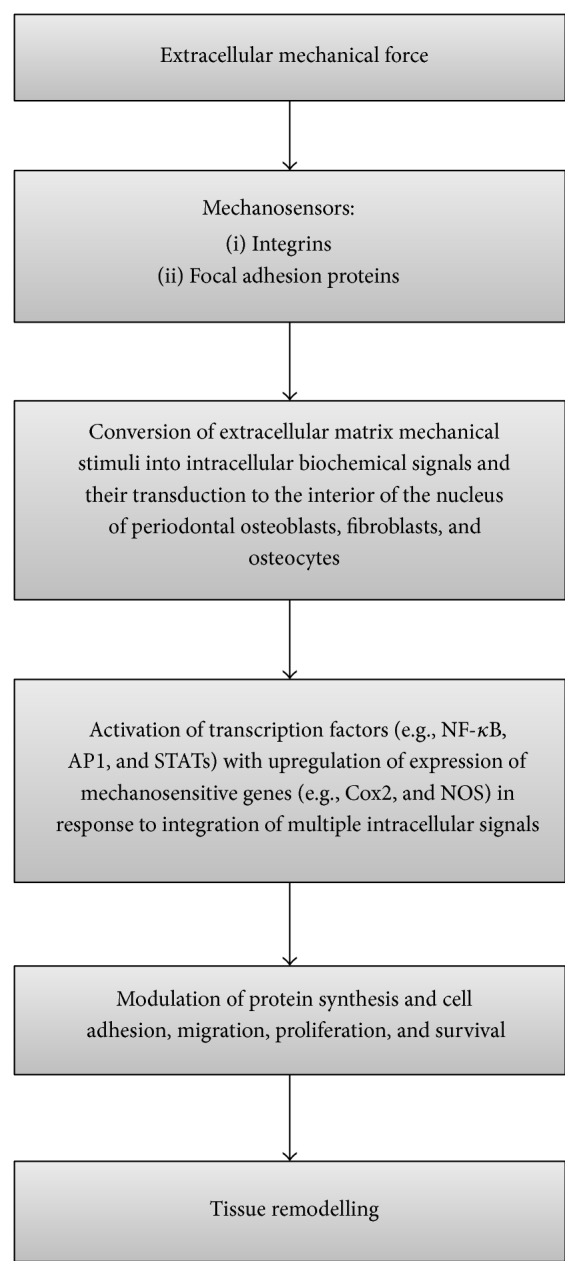
Flowchart showing the chain of events from application of mechanical forces to tissue remodelling.

Integrins and other proteins in focal adhesion domains act as cellular mechanosensors. Upon application of force, the stress that develops in the matrix and the consequent strain upon the mechanosensors triggers intracellular signals that control transcription factors determining gene expression. The activated genes regulate cell adhesion, migration, proliferation, apoptosis, and thus ultimately the response to the initial mechanical stress (Figure 2) [28, 31, 35–37]. Fibroblasts, osteoblasts, and osteocytes in the periodontium thus react to the applied loads of orthodontic treatment* via* the sequence of stress, strain including that related to fluid flow, and the production of biological mediators culminating in tissue remodelling [4].

**Figure 3:**
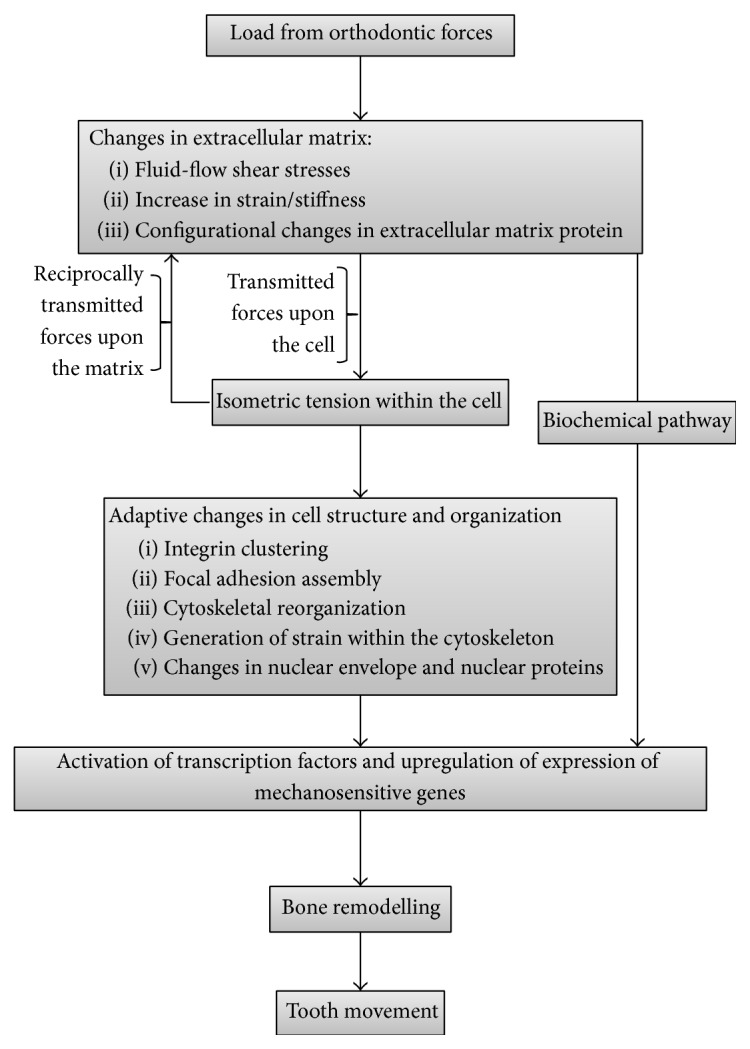
Flowchart showing some of the cell-matrix interactions inducing bone remodelling with consequent tooth movement.

Mechanical loads from application of orthodontic forces will stretch all the proteins of the extracellular matrix and the resulting configurational changes within the highly polymorphic protein structure can expose or hide molecular recognition sites, with inevitable cell activation and consequent modulation of cell responses [32, 35, 36]. In a stressed extracellular matrix, tensional forces exerted upon the cell by the matrix will cause intracellular isometric tension, further inducing configurational changes and exposure of hidden recognition molecules of some extracellular matrix proteins (Figure 3) [22, 31, 36, 37].

Therefore, a positive feedback loop is generated with extracellular matrix forces inducing intracellular stresses and regulating cell function and the intracellular cytoskeletally generated forces reciprocally influencing extracellular matrix biomechanics, function, and remodelling [38]. The strains developed in focal adhesions are thus generated by both intracellular contractile stresses and the stiffness of the extracellular matrix [22].

Intracellular strain resulting from stress in the extracellular matrix has the capacity to promote assembly of focal adhesions and clustering of integrins with their structural reinforcement and strengthening [35, 39] and to induce configurational alterations in some cytoskeletal filaments and crosslinks or in motor proteins, influencing intracellular signalling pathways (Figure 3). Furthermore, compression of the cell can cause intranuclear chromatin deformation with dysregulation of the transcriptional machinery and consequent alterations in protein production and secretion [23, 32]. The nature of the tissue changes will depend on the direction and magnitude of the applied force [39].

The cytoskeleton is connected to the nucleus through outer nuclear membrane proteins, the nesprins (Figure 1). In turn, nesprins interact with inner nuclear membrane proteins such as SUN1, SUN2, and Lamins [32]. Nuclear Lamins, specifically, are a class of intermediate filaments whose function is to support the mechanical integrity of the nucleus [25] and to bind to nuclear DNA, thus constituting the ultimate molecular link for transmission of mechanical load from the extracellular matrix to the nuclear proteins (Figure 1) [32].

## 5. Summary

Mechanotransduction is a complex dynamic process of converting biomechanical stimuli into intracellular biochemical signals eliciting tissue responses. In the local microenvironment of the periodontium, mechanical stresses from application of orthodontic loads cause intracellular changes within the cytoskeleton, configurational changes of extracellular matrix proteins, and the production of proinflammatory cytokines, all of which ultimately mediate bone resorption and formation, enabling the orthodontic load-induced tooth movement.
